# Investigation of the relationship between COVID-19 and pancreatic cancer using bioinformatics and systems biology approaches

**DOI:** 10.1097/MD.0000000000039057

**Published:** 2024-08-02

**Authors:** Chengxiang Fang, Haiyan Sun, Jing Wen, Xuehu Wu, Qian Wu, Dongsheng Zhai

**Affiliations:** a Department of Oncology, Minda Hospital of Hubei Minzu University, Enshi, P.R. China; b Department of Radiology, Maternal and Child Health Hospital of Enshi Tujia and Miao Autonomous Prefecture, Enshi, P.R. China; c Department of Hepatobiliary and Pancreatic Surgery, Minda Hospital of Hubei Minzu University, Enshi, P.R. China.

**Keywords:** COVID-19, differentially expressed genes, drug molecules, pancreatic cancer, protein–protein interaction

## Abstract

**Background:**

The coronavirus disease 2019 (COVID-19) pandemic, caused by the severe acute respiratory syndrome coronavirus 2 (SARS-CoV-2) virus, poses a huge threat to human health. Pancreatic cancer (PC) is a malignant tumor with high mortality. Research suggests that infection with SARS-CoV-2 may increase disease severity and risk of death in patients with pancreatic cancer, while pancreatic cancer may also increase the likelihood of contracting SARS-CoV-2, but the link is unclear.

**Methods:**

This study investigated the transcriptional profiles of COVID-19 and PC patients, along with their respective healthy controls, using bioinformatics and systems biology approaches to uncover the molecular mechanisms linking the 2 diseases. Specifically, gene expression data for COVID-19 and PC patients were obtained from the Gene Expression Omnibus datasets, and common differentially expressed genes (DEGs) were identified. Gene ontology and pathway enrichment analyses were performed on the common DEGs to elucidate the regulatory relationships between the diseases. Additionally, hub genes were identified by constructing a protein–protein interaction network from the shared DEGs. Using these hub genes, we conducted regulatory network analyses of microRNA/transcription factors-genes relationships, and predicted potential drugs for treating COVID-19 and PC.

**Results:**

A total of 1722 and 2979 DEGs were identified from the transcriptome data of PC (GSE119794) and COVID-19 (GSE196822), respectively. Among these, 236 common DEGs were found between COVID-19 and PC based on protein–protein interaction analysis. Functional enrichment analysis indicated that these shared DEGs were involved in pathways related to viral genome replication and tumorigenesis. Additionally, 10 hub genes, including extra spindle pole bodies like 1, holliday junction recognition protein, marker of proliferation Ki-67, kinesin family member 4A, cyclin-dependent kinase 1, topoisomerase II alpha, cyclin B2, ubiquitin-conjugating enzyme E2 C, aurora kinase B, and targeting protein for Xklp2, were identified. Regulatory network analysis revealed 42 transcription factors and 23 microRNAs as transcriptional regulatory signals. Importantly, lucanthone, etoposide, troglitazone, resveratrol, calcitriol, ciclopirox, dasatinib, enterolactone, methotrexate, and irinotecan emerged as potential therapeutic agents against both COVID-19 and PC.

**Conclusion:**

This study unveils potential shared pathogenic mechanisms between PC and COVID-19, offering novel insights for future research and therapeutic strategies for the treatment of PC and SARS-CoV-2 infection.

## 1. Introduction

Coronavirus disease 2019 (COVID-19) is a respiratory illness caused by the severe acute respiratory syndrome coronavirus 2 (SARS-CoV-2).^[1]^ The disease ranges in severity from mild symptoms, such as fever and cough, to severe cases that can lead to acute respiratory distress syndrome, septic shock, refractory metabolic acidosis, and coagulation disorders.^[2]^ SARS-CoV-2 is composed of several structural proteins: nucleocapsid protein, envelope protein, membrane protein, and spike protein.^[3]^ The spike protein is particularly critical as it facilitates the virus’s entry into host cells by binding to the angiotensin-converting enzyme 2 (ACE2) receptor on the surface of these cells.^[4]^ ACE2 receptors are widely distributed throughout the body, including in the lungs, heart, kidneys, intestines, blood vessels, liver, testicles, gallbladder, and pancreas. This widespread presence of ACE2 explains why COVID-19 can affect multiple organ systems, making it a systemic disease.^[5]^ The systemic nature of COVID-19 can lead to a variety of complications beyond respiratory issues, such as cardiovascular, renal, gastrointestinal, and hepatic problems.^[6]^ Thus, it is imperative to investigate the mechanisms by which COVID-19 interacts with other diseases, as this knowledge could reveal potential therapeutic targets and inform strategies to mitigate the multifaceted impacts of the virus. Understanding these interactions could also improve clinical management and outcomes for patients with comorbid conditions.

Pancreatic cancer (PC) is a malignant tumor that originates in the tissues of the pancreas.^[7]^ The pancreas, located behind the stomach, is a crucial digestive organ responsible for secreting digestive enzymes and hormones such as insulin, which are essential for regulating blood sugar levels and aiding in the digestion of food.^[8]^ The symptoms of pancreatic cancer vary depending on the tumor’s location and size. Common symptoms include abdominal pain, typically in the upper abdomen and sometimes radiating to the back, jaundice, which is characterized by yellowing of the skin and eyes due to bile duct obstruction, weight loss due to decreased appetite and malabsorption of nutrients, and digestive issues such as indigestion and nausea.^[9]^ The main treatments for pancreatic cancer are surgery, radiation therapy, and chemotherapy. Surgery is often considered the best option for a potential cure, especially in cases where the cancer is detected early and is confined to the pancreas.^[10]^ The prognosis for pancreatic cancer is often poor due to the lack of early symptoms and its tendency to metastasize. Typically diagnosed at an advanced stage, treatment becomes more challenging. As a result, the 5-year survival rate is low, around 10%, but this varies with the stage at diagnosis and treatment effectiveness. Early detection and treatment can significantly improve prognosis, underscoring the need for ongoing research and advancements in diagnostics and therapies.^[11]^ Moreover, the level of ACE2 protein in pancreatic cancer tissues was higher than that in adjacent nontumor tissues,^[12]^ suggesting that pancreatic cancer patients were more susceptible to SARS-CoV-2 infection. Malignancy is reported to be associated with a poorer prognosis in patients with COVID-19, with a 2-fold increased chance of severity and mortality.^[13]^

Therefore, given the COVID-19 epidemic and the high prevalence of pancreatic cancer, the findings of this study are particularly relevant. In order to elucidate the relationship between pancreatic cancer and COVID-19, 2 datasets were analyzed. GSE196822 and GSE119794 were utilized for COVID-19 and pancreatic cancer, respectively, and were sourced from the Gene Expression Omnibus (GEO) database. Differentially expressed genes (DEGs) were identified within each dataset, and the shared DEGs for COVID-19 and PC were determined. Subsequent analyses, including functional annotation and transcriptional regulator analyses, were performed to enhance our understanding of the potential mechanism. Finally, drugs were predicted based on these findings.

## 2. Materials and methods

### 2.1. Sources of gene expression datasets

To explore the potential connection among COVID-19 and PC, RNA-seq datasets were acquired from the GEO database of the NCBI (https://www.ncbi.nlm.nih.gov/geo/). The GEO accession ID of COVID-19 is GSE196822,^[14]^ which was transcriptome of the whole blood of 34 COVID-19 patients and 9 healthy controls. The GSE196822 dataset was established by high-throughput sequencing Illumina HiSeq 4000 (Homo sapiens). Besides, the GEO accession ID of PC is GSE119794,^[15]^ which was processed by a high-throughput sequencing system called Illumina HiSeq 2000 (Homo sapiens). The GSE119794 dataset consists of 10 paired tumor and normal pancreatic samples from pancreatic cancer patients.

### 2.2. Identification of DEGs and shared DEGs among COVID-19 and PC

Correct identification of DEGs under specific conditions is key to understanding phenotypic variation.^[16]^ The DEGs among COVID-19 and PC were screened out by DEseq2 package^[17]^ in R software (version 4.2.2, https://www.R-project.org/), respectively. Genes with |log_2_ Fold Change|≥1 and *P* value < .05 were identified as DEGs. An online VENN graph mapping platform named Jvenn^[18]^ (http://jvenn.toulouse.inra.fr/app/example.html) was applied to acquire common DEGs of COVID-19 and PC.

### 2.3. Annotation of the gene ontology and pathways of shared DEGs among COVID-19 and PC

Functional enrichment analysis is a common method for characterizing differentially expressed genes based on gene ontology terms, pathways, and other annotated gene sets. Constructed by biological process (BP), cellular component (CC), and molecular function, Gene Ontology (GO) is a classification system of gene function aiming at describing the properties of genes and gene products.^[19]^ The Reactome is a database that supports basic research, genome analysis, modeling, systems biology, and education.^[20]^ The BioPlanet is a comprehensive informatics resource that classifies all pathways, their health and disease state annotations, the targets within them, and the relationships between them.^[21]^ The Kyoto Encyclopedia of Genes and Genomes (KEGG) is a database resource for understanding the advanced functions and utility of biological systems, such as cells, organisms, and ecosystems, from molecular-level information, particularly large-scale molecular datasets generated by genome sequencing and other high-throughput experimental techniques.^[22]^ The WikiPathways is a comprehensive database concerning biological knowledge in pathways.^[23]^ To investigate the functions of common DEGs, the analysis of GO, Reactome, BioPlanet, KEGG, and WikiPathways was conducted utilizing Enrichr^[24]^ (http://amp.pharm.mssm.edu/Enrichr), a wide range of online gene set enrichment tool. The *P* value < .05 was used as a criterion to screen for reliable results.

### 2.4. Construction of protein–protein interaction network

Proteins are the most critical elements of an organism’s biological function; they do not perform their functions alone, but interact with other proteins and molecules.^[25]^ STRING (https://www.string-db.org/) (11.5 version) is a database designed to integrate all known and predicted associations between proteins, including physical interactions and functional associations.^[26]^ The common DEGs were analyzed, and the PPI network was constructed with a comprehensive score >0.4 in this research. Further experimental studies of the PPI network were then conducted using Cytoscape (version 3.9).^[27]^

### 2.5. Extraction of hub genes

Based on the PPI network, the CytoHubba (https://apps.cytoscape.org/apps/cytohubba), the plugin of Cytoscape, was utilized to select the hub genes. Cytohubba is a significant Cytoscape application, which can rank and extract crucial, potential, or targeted components of a biological network.^[28]^ Furthermore, Cytohubba offers 11 methods for exploring networks from diverse perspectives, with maximal clique centrality emerging as the most effective among them.^[28]^ The maximal clique centrality feature within Cytohubba was utilized to validate the top 10 hub genes within the PPI network.

### 2.6. Identification of transcription factor and microRNA

A transcription factor (TF) is a protein that regulates the process and rate of DNA transcription by binding to specific DNA sequences.^[29]^ MicroRNA (miRNA) is a small, single-stranded RNA molecule that pairs with complementary mRNA sequences, leading to their cleavage, destabilization, and subsequent gene silencing.^[30]^ Both of these molecules play crucial roles in modulating gene expression. JASPAR (http://jaspar.genereg.net) is a database storing manually curated TF binding profiles as position frequency matrices.^[31]^ Position frequency matrices summarize occurrences of each nucleotide at each position in a set of observed TF-DNA interactions and are capable of being put into practice to scan any DNA sequence to predict TF binding sites.^[32]^ Via the tool of NetworkAnalyst,^[33]^ we identified topologically feasible TFs from the JASPAR database and associated them with hub genes. MiRTarBase is a well-known miRNA-target interaction comprehensive database.^[34]^ Using NetworkAnalyst, we identified miRNAs interacting with hub genes from MiRTarBase.

### 2.7. Evaluation of candidate drugs

In this analysis, protein–drug interactions and potential pharmacological molecules were predicted based on the hub genes between COVID-19 and PC. Drug Signatures Database^[35]^ is a database that collects a large number of drug gene expression characteristics. These signatures are based on data on changes in gene expression after the drug is treated, helping researchers understand the drug’s mechanism of action.

## 3. Results

### 3.1. Identification of DEGs and common DEGs among COVID-19 and PC

**Figure 1.**
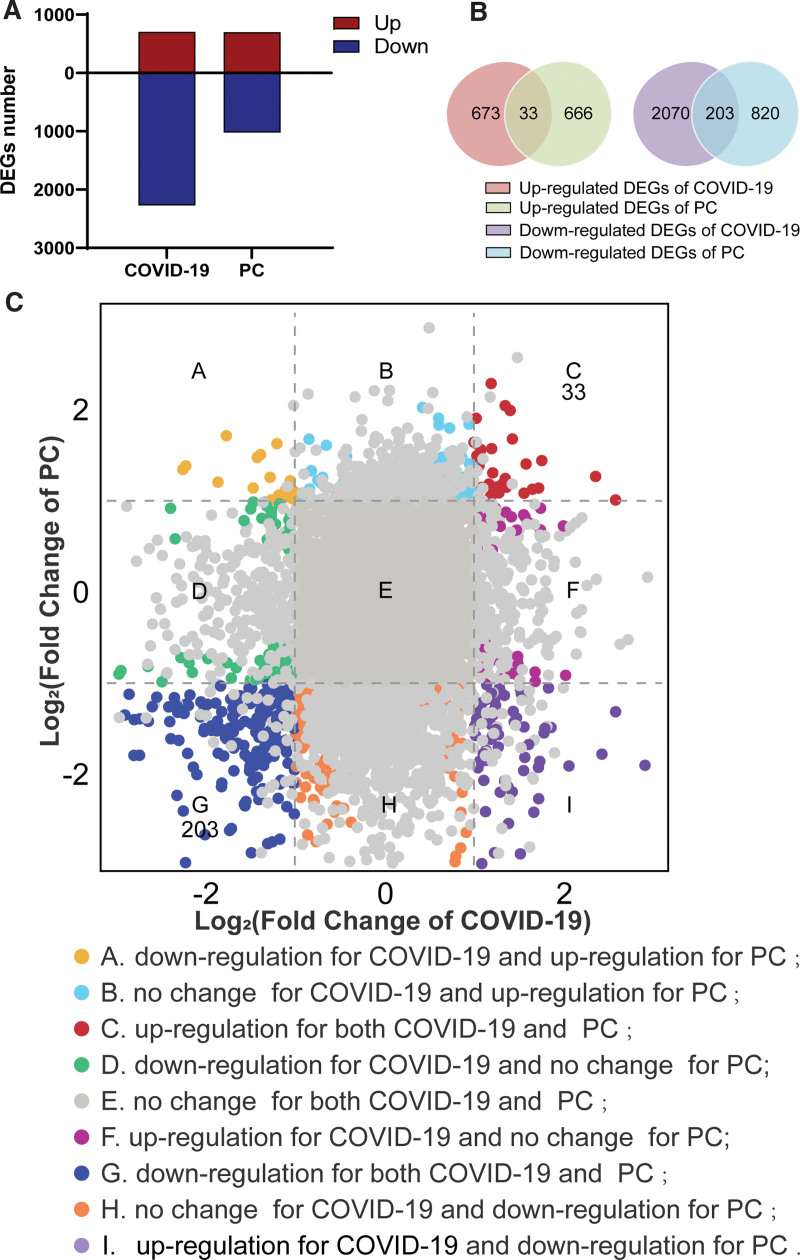
Transcriptional regulatory of COVID-19 and PC. (A) Number of DEGs (|log_2_ Fold Change|≥1 and *P* value < .05) at transcriptional level. The red and blue bars refer to the number of up-regulated and down-regulated DEGs, respectively. (B) The Venn diagram showing the common DEGs between COVID-19 and PC. (C) Fold changes of COVID-19 and PC at transcriptional level. Nine categories in different colors indicate 9 responsive groups (|log_2_ Fold Change|≥1 and *P* value < .05). COVID-19|=|coronavirus disease 2019, DEGs|=|differentially expressed genes, PC|=|pancreatic cancer.

Aiming to reveal the interrelationship between COVID-19 and PC, we identified the DEGs of COVID-19 and PC, respectively. For COVID-19 dataset, there are 2979 DEGs, including 706 up-regulated DEGs and 2273 down-regulated DEGs. Similarly, we detected 1722 DEGs between paired tumor and normal pancreatic samples from PC patients, with 699 up-regulated DEGs and 1023 down-regulated DEGs included. Figure 1A and Table 1 counted the number of differentially expressed genes in COVID-19 and PC. Besides, cross-comparative analysis revealed COVID-19 and PC shared 236 DEGs, 33 up-regulated in both datasets and 203 down-regulated in both datasets (Fig. 1B). Based on the |log_2_ Fold Change|≥1 and *P* value < .05 as cutoff, genes were classified into 9 categories (Fig. 1C). The accordant groups (category C and G) showed the same directionality of 236 common DEGs. Therefore, by comparing transcriptional maps, we found a transcriptional regulatory correlation between COVID-19 and PC.

**Table 1:** Overview of the datasets and DEGs.

Disease name	GEO accession	GEO platform	Total DEGs	Up-regulated DEGs	Down-regulated DEGs
COVID-19	GSE196822	GPL20301	2979	706	2273
PC	GSE119794	GPL11154	1722	699	1023

COVID-19 = coronavirus disease 2019, DEGs = differentially expressed genes, PC = pancreatic cancer.

### 3.2. Gene ontology and pathway enrichment analysis of shared DEGs among COVID-19 and PC

**Figure 2.**
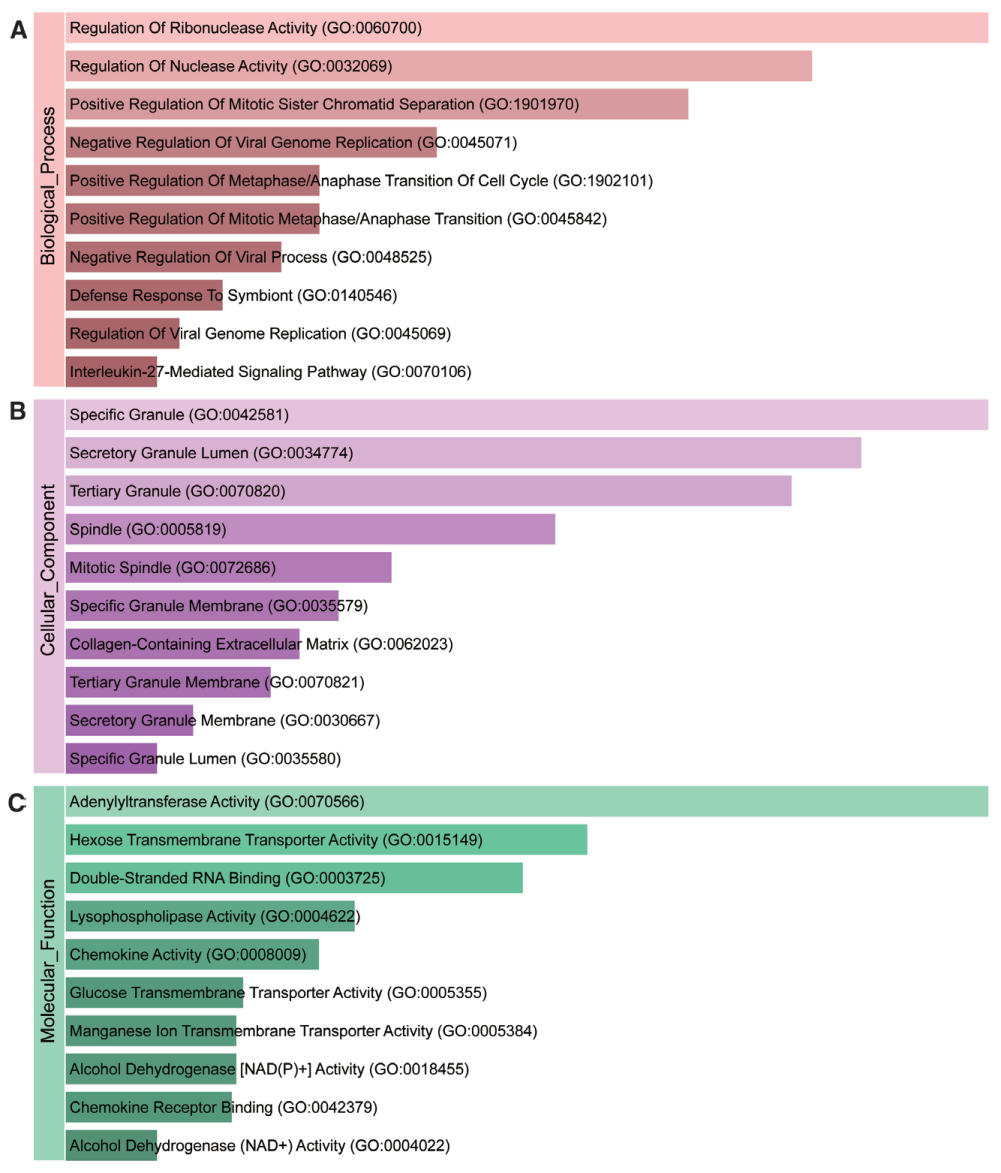
The bar charts of GO analysis of shared DEGs between COVID-19 and PC. (A) Biological progress. (B) Cellular component. (C) Molecular function. COVID-19|=|coronavirus disease 2019, DEGs|=|differentially expressed genes, GO|=|gene ontology, PC|=|pancreatic cancer.

To investigate the potential functions of shared DEGs, we used the Enrichr tool to perform gene ontology and pathway enrichment analysis. Figure 2 and Table 2 depict the top 10 terms of GO analysis, including BP, CC, and molecular function. The GO analysis reveals that pathways associated with viral genome replication and cancer development are significantly enriched, including Negative Regulation of Viral Genome Replication (GO:0045071), Negative Regulation of Viral Process (GO:0048525), Defense Response to Symbiont (GO:0140546), Regulation of Viral Genome Replication (GO:0045069), Positive Regulation of Mitotic Sister Chromatid Separation (GO:1901970), Positive Regulation of Metaphase/Anaphase Transition of Cell Cycle (GO:1902101), Positive Regulation of Mitotic Metaphase/Anaphase Transition (GO:0045842), Mitotic Spindle (GO:0072686), Interleukin-27-Mediated Signaling Pathway (GO:0070106), Chemokine Activity (GO:0008009), and Chemokine Receptor Binding (GO:0042379).

**Table 2:** GO analysis of overlapped DEGs among COVID-19 and PC.

Category	Term	*P* value	Genes
Biological progress	Regulation of Ribonuclease Activity (GO:0060700)	9.36E-08	OAS1/OAS2/OAS3/OASL
	Regulation of Nuclease Activity (GO:0032069)	2.78E-07	OAS1/OAS2/OAS3/OASL
	Positive Regulation of Mitotic Sister Chromatid Separation (GO:1901970)	5.98E-07	CDC20/ESPL1/UBE2C/AURKB/DLGAP5
	Negative Regulation of Viral Genome Replication (GO:0045071)	2.83E-06	RSAD2/OAS1/SLPI/OAS2/OAS3/ISG15/OASL
	Positive Regulation of Metaphase/Anaphase Transition of Cell Cycle (GO:1902101)	5.84E-06	CDC20/ESPL1/UBE2C/DLGAP5
	Positive Regulation of Mitotic Metaphase/Anaphase Transition (GO:0045842)	5.84E-06	CDC20/ESPL1/UBE2C/DLGAP5
	Negative Regulation of Viral Process (GO:0048525)	7.39E-06	RSAD2/OAS1/SLPI/OAS2/OAS3/ISG15/OASL
	Defense Response to Symbiont (GO:0140546)	1.06E-05	RSAD2/IFI27/OAS1/OAS2/OAS3/IFI6/F2RL1/ISG15/DDX60/OASL
	Regulation of Viral Genome Replication (GO:0045069)	1.39E-05	RSAD2/OAS1/SLPI/OAS2/OAS3/ISG15/OASL
	Interleukin-27-Mediated Signaling Pathway (GO:0070106)	1.59E-05	OAS1/OAS2/OASL
Cellular component	Specific Granule (GO:0042581)	5.43E-08	AOC1/ANXA3/PLAUR/MCEMP1/SLC2A5/OSCAR/CEACAM1/SLPI/PLAU/TCN1/CLEC5A/LCN2/ADAM8
	Secretory Granule Lumen (GO:0034774)	2.34E-07	CDA/ECM1/AOC1/SERPINA1/FN1/PPBP/UNC13D/F5/OSCAR/HK3/PKM/SLPI/TCN1/LCN2/S100P/ALDOA/S100A11
	Tertiary Granule (GO:0070820)	5.21E-07	CDA/CEACAM1/PLAU/TCN1/SLC11A1/CLEC5A/MCEMP1/ADAM8/PPBP/ALDOA/CD55/OSCAR
	Spindle (GO:0005819)	7.86E-06	CDC20/TPX2/ESPL1/KIFC1/CKAP2L/KIF4A/CDK1/CAPG/HMMR/SKA3/DLGAP5/AURKB
	Mitotic Spindle (GO:0072686)	5.16E-05	TPX2/KIF18B/ESPL1/CKAP2L/KIFC1/CDK1/CAPG/SKA3/DLGAP5
	Specific Granule Membrane (GO:0035579)	9.48E-05	CEACAM1/PLAU/CLEC5A/PLAUR/MCEMP1/ADAM8/SLC2A5
	Collagen-Containing Extracellular Matrix (GO:0062023)	1.48E-04	COL17A1/ACHE/ECM1/SERPINA1/F12/COL12A1/FN1/COL19A1/FBLN5/PKM/SLPI/COL7A1/S100A6/CLC
	Tertiary Granule Membrane (GO:0070821)	2.07E-04	CEACAM1/PLAU/SLC11A1/CLEC5A/MCEMP1/ADAM8
	Secretory Granule Membrane (GO:0030667)	5.03E-04	CEACAM1/VNN1/CEACAM6/PLAU/SLC11A1/CLEC5A/PLAUR/MCEMP1/ADAM8/SLC2A5/CD55
	Specific Granule Lumen (GO:0035580)	7.62E-04	AOC1/SLPI/TCN1/LCN2/OSCAR
Molecular function	Adenylyltransferase Activity (GO:0070566)	1.97E-04	OAS1/OAS2/OAS3/OASL
	Hexose Transmembrane Transporter Activity (GO:0015149)	8.11E-04	SLC2A1/PPBP/SLC2A5
	Double-Stranded RNA Binding (GO:0003725)	0.001018	OAS1/OAS2/OAS3/DDX60/OASL
	Lysophospholipase Activity (GO:0004622)	0.001844	GDPD3/CLC/MGLL
	Chemokine Activity (GO:0008009)	0.002091	KLF5/XCL2/XCL1/PPBP
	Glucose Transmembrane Transporter Activity (GO:0005355)	0.002734	SLC2A1/PPBP/SLC2A5
	Manganese Ion Transmembrane Transporter Activity (GO:0005384)	0.0028	SLC11A1/ATP2C2
	Alcohol Dehydrogenase [NAD(P)+] Activity (GO:0018455)	0.0028	DHRS9/ADHFE1
	Chemokine Receptor Binding (GO:0042379)	0.002845	KLF5/XCL2/XCL1/PPBP
	Alcohol Dehydrogenase (NAD+) Activity (GO:0004022)	0.003705	DHRS9/ADHFE1

COVID-19 = coronavirus disease 2019, DEGs = differentially expressed genes, GO = gene ontology, KIF4A = kinesin family member 4A, PC = pancreatic cancer.

**Figure 3.**
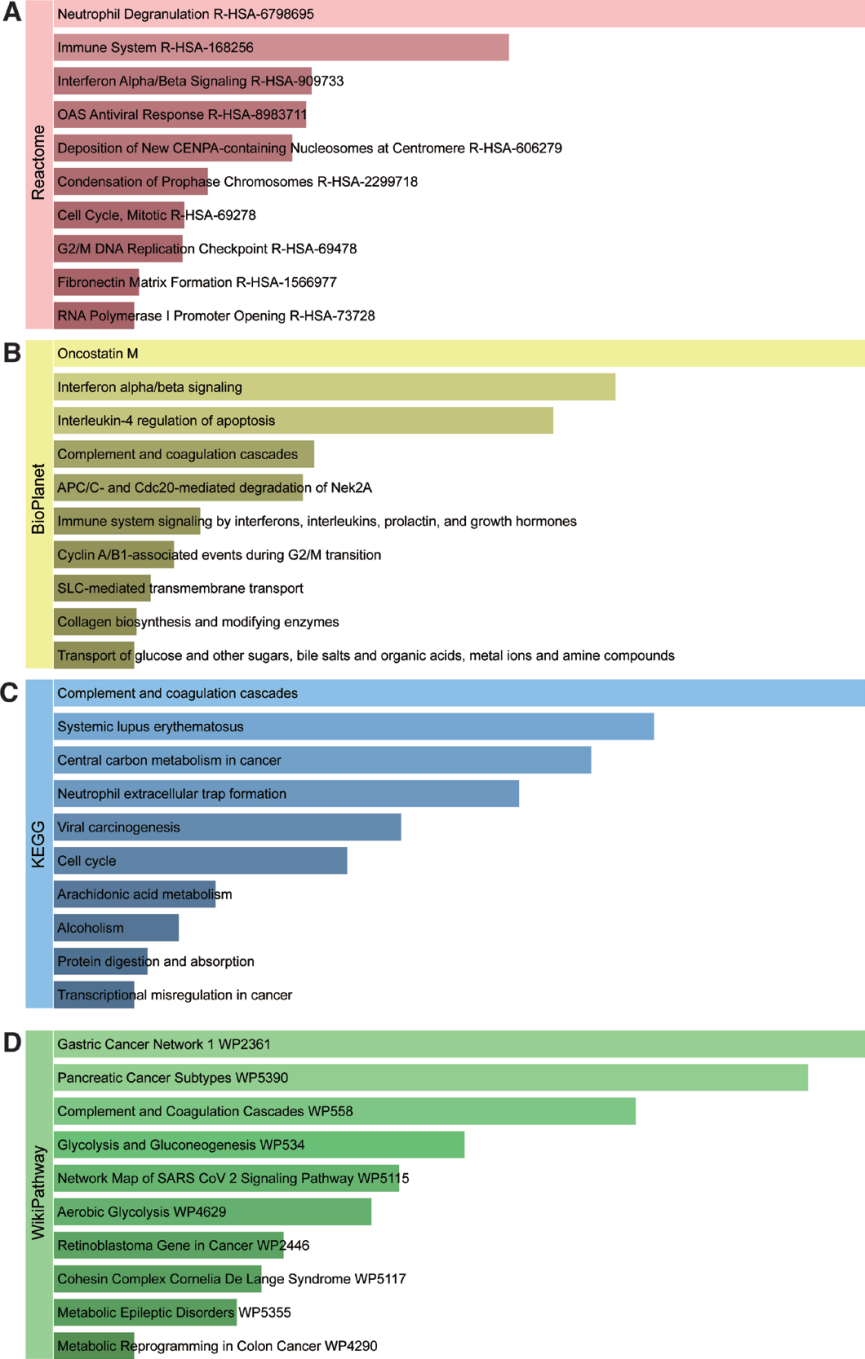
The bar charts of pathway enrichment analysis of overlapped DEGs among COVID-19 and PC. (A) Reactome. (B) BioPlanet. (C) KEGG. (D) WikiPathway. COVID-19|=|coronavirus disease 2019, KEGG|=|Kyoto encyclopedia of genes and genomes, PC|=|pancreatic cancer.

To identify the most significant pathways of the mutual DEGs, we conducted an enrichment analysis utilizing Reactome, BioPlanet, KEGG, and WikiPathway databases. The top 10 terms in the 4 databases are listed in Table 3. Figure 3 shows the particular information of the pathways. The Reactome enrichment analysis showed that the shared DEGs are mainly involved in Immune System (R-HSA-168256), Interferon Alpha/Beta Signaling (R-HSA-909733), OAS Antiviral Response (R-HSA-8983711), Cell Cycle, Mitotic (R-HSA-69278), and G2/M DNA Replication Checkpoint (R-HSA-69478). Furthermore, the Bioplanet analysis showed the shared DEGs may influence Oncostatin M, Interferon Alpha/Beta Signaling, Interleukin-4 regulation of apoptosis, Immune system signaling by interferons, interleukins, prolactin, and growth hormones, and Cyclin A/B1-associated events during G2/M transition. The most significant terms in KEGG are Central carbon metabolism in cancer, Neutrophil extracellular trap formation, Viral carcinogenesis, Cell cycle, and Transcriptional misregulation in cancer. Most important of all, the WikiPathways analysis revealed mutual DEGs significantly enriched in the Pancreatic Cancer Subtypes (WP5390), and Network Map of SARS-CoV-2 Signaling Pathway (WP5115). These results strongly suggest that these common DEGs are involved in pathways associated with viral genome replication and tumorigenesis.

**Table 3:** Pathway enrichment analysis of common DEGs between COVID-19 and PC.

Category	Pathways	*P* value	Genes
Reactome	Neutrophil Degranulation R-HSA-6798695	3.43E-10	CDA/SERPINA1/SLC2A5/ABCA13/LOC112694756/PLAU/CLEC5A/S100A11/CD55/AOC1/SLC11A1/PLAUR/MCEMP1/PPBP/UNC13D/OSCAR/CEACAM1/VNN1/PKM/SLPI/TCN1/CEACAM6/LCN2/ADAM8/S100P
	Immune System R-HSA-168256	9.32E-08	CDA/IL1RN/SERPINA1/IL1RAP/SLC2A5/ABCA13/LOC112694756/OASL/CDC20/PLAU/CLEC5A/FCER1A/CTSE/RSAD2/SLC11A1/IL1R2/PLAUR/MCEMP1/UNC13D/OSCAR/CEACAM1/PKM/IFI27/SLPI/OAS1/CEACAM6/OAS2/OAS3/KLRF1/ADAM8/TRIM10/KLRB1/IFI6/CD1C/PI3/XDH/S100A11/CD55/VASP/AOC1/SIGLEC12/UBE2C/FN1/ISG15/PPBP/VNN1/TCN1/KIF4A/LCN2/S100P
	Interferon Alpha/Beta Signaling R-HSA-909733	2.09E-06	RSAD2/IFI27/OAS1/OAS2/OAS3/IFI6/ISG15/OASL
	OAS Antiviral Response R-HSA-8983711	2.27E-06	OAS1/OAS2/OAS3/OASL
	Deposition of New CENP-A-containing Nucleosomes at Centromere R-HSA-606279	2.83E-06	H2BC5/H2BC21/H2BC11/H2BC4/H2AC19/HJURP/KNL1
	Condensation of Prophase Chromosomes R-HSA-2299718	1.07E-05	H2BC5/H2BC21/H2BC4/H2BC11/H2AC19/CDK1
	Cell Cycle, Mitotic R-HSA-69278	1.55E-05	TOP2A/H2BC5/UBE2C/CDCA5/H2BC4/H2AC19/KNL1/HMMR/PKMYT1/AURKB/CDC20/TPX2/CCNB2/CDC45/ESPL1/H2BC21/H2BC11/CDK1/NEK2
	G2/M DNA Replication Checkpoint R-HSA-69478	1.59E-05	CCNB2/CDK1/PKMYT1
	Fibronectin Matrix Formation R-HSA-1566977	3.16E-05	CEACAM1/CEACAM6/FN1
	RNA Polymerase I Promoter Opening R-HSA-73728	3.40E-05	H2BC5/H2BC21/H2BC4/H2BC11/H2AC19
Bioplanet	Oncostatin M	9.65E-07	CDA/ECM1/CRABP2/ANXA3/FN1/PLAUR/OAS1/TCN1/CEACAM6/PLAU/LCN2/S100P/PI3/XDH/SLC16A3/MGLL
	Interferon alpha/beta signaling	1.02E-05	IFI27/OAS1/OAS2/OAS3/IFI6/ISG15/OASL
	Interleukin-4 regulation of apoptosis	1.84E-05	TOP2A/IL1RN/RSAD2/LRRN3/FN1/IFI6/INHBA/CD1C/CDC45/XCL1/S100P/MXD1/MET
	Complement and coagulation cascades	1.77E-04	SERPINA1/PLAU/F12/PLAUR/CD55/F5
	APC/C- and Cdc20-mediated degradation of Nek2A	1.97E-04	CDC20/UBE2C/CDK1/NEK2
	Immune system signaling by interferons, interleukins, prolactin, and growth hormones	5.19E-04	IL1RN/IFI27/OAS1/OAS2/IL1R2/OAS3/IFI6/CDK1/ISG15/IL1RAP/OASL
	Cyclin A/B1-associated events during G2/M transition	6.65E-04	CCNB2/CDK1/PKMYT1
	SLC-mediated transmembrane transport	8.31E-04	HK3/SLCO4A1/SLC11A1/SLC22A18/SLC4A10/SLC2A1/SLC6A12/SLC16A10/SLC2A5/SLC16A3
	Collagen biosynthesis and modifying enzymes	9.49E-04	COL17A1/COL7A1/COL12A1/PLOD2/COL19A1
KEGG	Complement and coagulation cascades	5.09E-04	SERPINA1/PLAU/F12/PLAUR/CD55/F5
	Systemic lupus erythematosus	0.001123477	H4C8/H2BC5/H2BC21/H2BC11/H2BC4/H2AC19/H2AC18
	Central carbon metabolism in cancer	0.001422579	HK3/PKM/SLC2A1/SLC16A3/MET
	Neutrophil extracellular trap formation	0.001865235	H4C8/H2BC5/H2BC21/AQP9/H2BC11/H2BC4/H2AC19/H2AC18
	Viral carcinogenesis	0.002903171	CDC20/H4C8/PKM/H2BC5/H2BC21/H2BC4/H2BC11/CDK1
	Cell cycle	0.003558454	CDC20/CCNB2/CDC45/ESPL1/CDK1/PKMYT1
	Arachidonic acid metabolism	0.005829498	GPX2/EPHX2/CYP4F3/PTGES
	Alcoholism	0.006693872	H4C8/H2BC5/H2BC21/H2BC11/H2BC4/H2AC19/H2AC18
	Protein digestion and absorption	0.007528215	COL17A1/COL7A1/COL12A1/SLC16A10/COL19A1
	Transcriptional misregulation in cancer	0.007910907	ETV7/PAX8/BCL2A1/HPGD/PLAU/IL1R2/MET
WikiPathways	Gastric Cancer Network 1 WP2361	1.43E-05	TOP2A/TPX2/UBE2C/S100P/E2F7
	Pancreatic Cancer Subtypes WP5390	2.05E-05	DHRS9/CEACAM6/SLC2A1/TFF3/CTSE/FAM83A
	Complement and Coagulation Cascades WP558	6.14E-05	SERPINA1/PLAU/F12/PLAUR/CD55/F5
	Glycolysis and Gluconeogenesis WP534	1.82E-04	HK3/PKM/SLC2A1/SLC2A5/ALDOA
	Network Map of SARS-CoV-2 Signaling Pathway WP5115	2.76E-04	CCNB2/IFI27/OAS2/IL1R2/COL7A1/FN1/IFI6/CDK1/APOL1/FAM83A
	Aerobic Glycolysis WP4629	3.30E-04	PKM/SLC2A1/ALDOA
	Retinoblastoma Gene in Cancer WP2446	5.77E-04	TOP2A/CCNB2/ANLN/CDC45/KIF4A/CDK1
	Cohesin Complex Cornelia De Lange Syndrome WP5117	6.64E-04	ESPL1/CDCA5/CDK1/AURKB
	Metabolic Epileptic Disorders WP5355	7.76E-04	HK3/PKM/SLC2A1/SLC2A5/ALDOA/XDH
	Metabolic Reprogramming in Colon Cancer WP4290	0.001488095	HK3/PKM/SLC2A1/SLC16A3

COVID-19 = coronavirus disease 2019, DEGs = differentially expressed genes, KEGG= Kyoto Encyclopedia of Genes and Genomes, KIF4A = kinesin family member 4A, PC = pancreatic cancer.

### 3.3. Construction of PPI network and identification of hub gene

**Figure 4.**
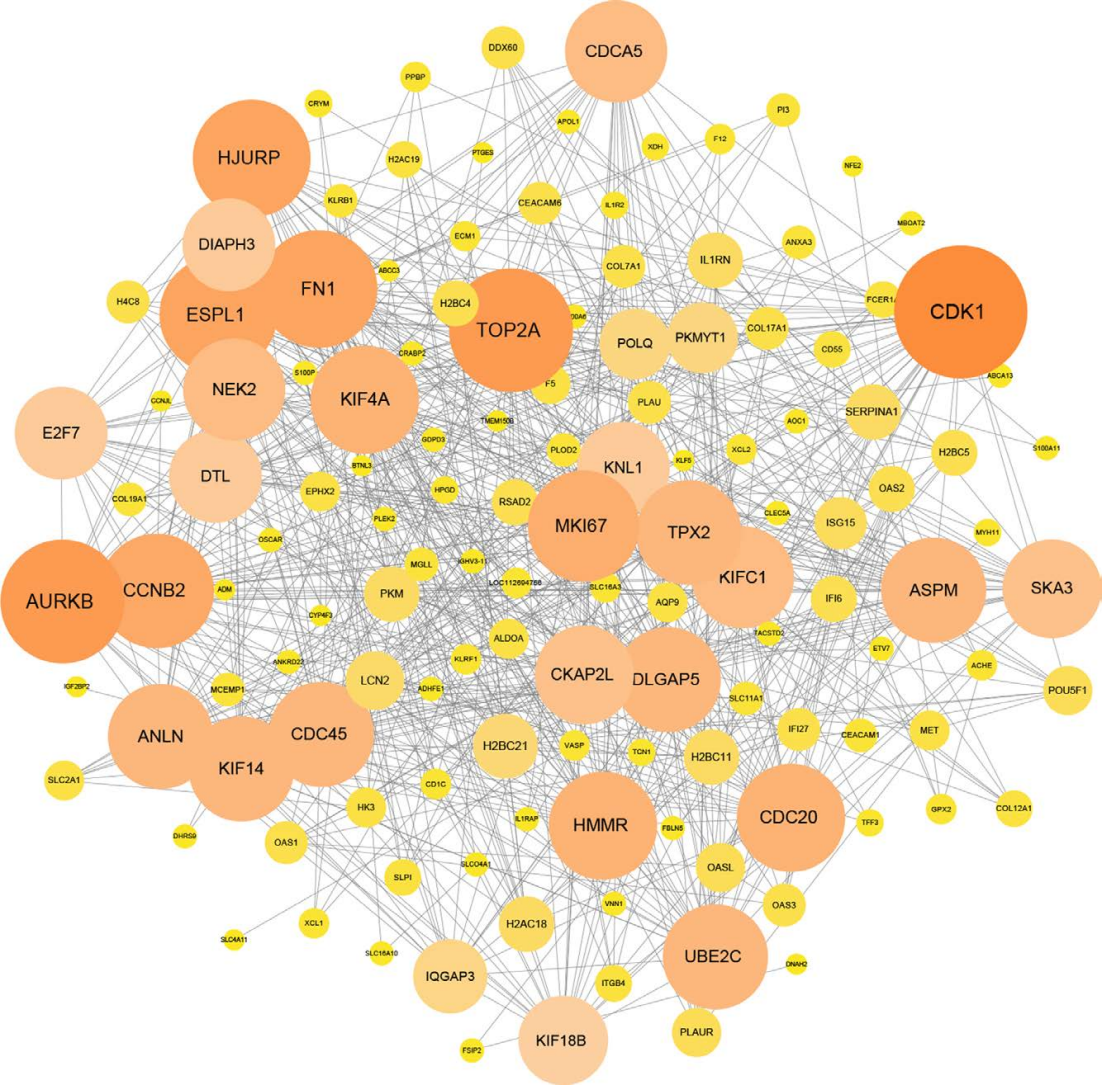
PPI network of the mutual DEGs between COVID-19 and PC. The round nodes represent mutual DEGs-encoded proteins, and the edges symbolize the interaction of the nodes. The size of each node represents the amount of its interactivity. The PPI network contains 677 edges and 133 nodes. COVID-19|=|coronavirus disease 2019, DEGs|=|differentially expressed genes, PC|=|pancreatic cancer, PPI|=|protein–protein interaction.

**Figure 5.**
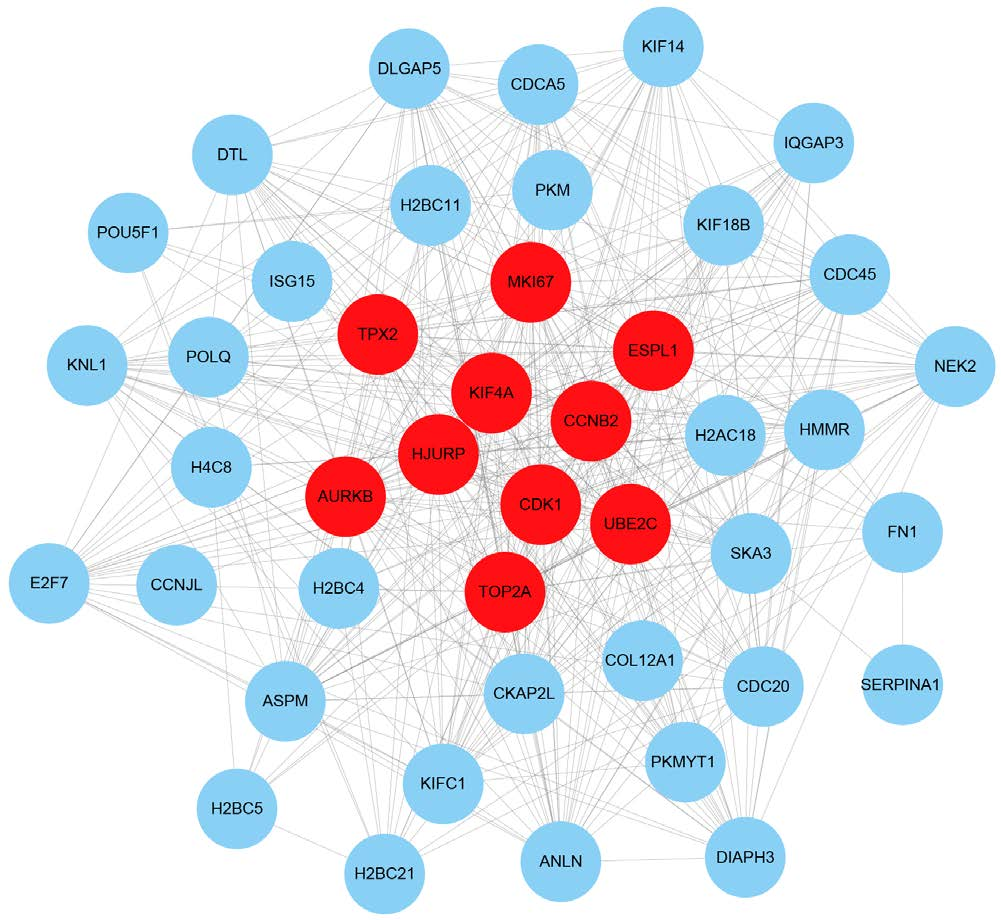
The hub genes were identified from the PPI network using the Cytohubba plug in Cytoscape. Here, the colored nodes represent the selected top 10 hub genes and their interactions with other molecules. The network has 451 edges and 43 nodes. PPI|=|protein–protein interaction.

A network of interactions between 236 overlapping DEGs-encoded proteins, consisting of 677 edges and 133 nodes, built by the STRING database, was visualized using the Cytoscape 3.10.2 tool (Fig. 4). The Cytohubba plugin was then used to identify the 10 genes with the highest degree value in this network, with these genes (extra spindle pole bodies like 1 [ESPL1], holliday junction recognition protein [HJURP], marker of proliferation Ki-67 [MKI67], kinesin family member 4A (KIF4A), cyclin-dependent kinase 1 [CDK1], topoisomerase II alpha [TOP2A], cyclin B2 [CCNB2], ubiquitin-conjugating enzyme E2 C [UBE2C], aurora kinase B [AURKB], and targeting protein for Xklp2 [TPX2]) being identified as hub genes (Fig. 5).

### 3.4. Identification of regulatory networks at transcriptional level

**Figure 6.**
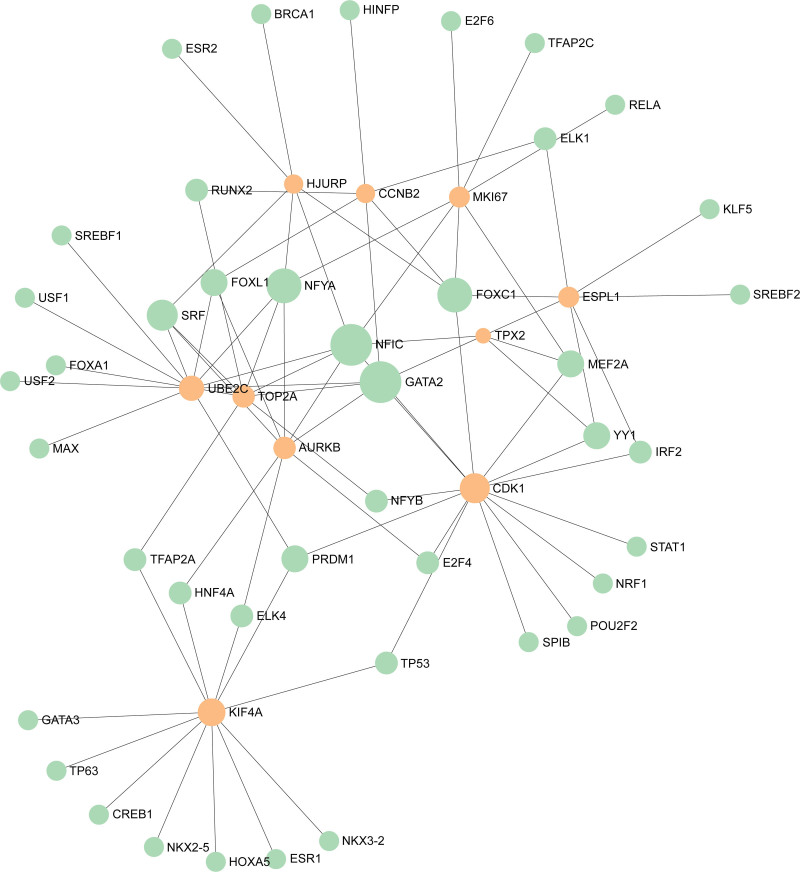
The construction of a regulatory interaction network of hub genes and TFs. In this figure, orange circles represent hub genes, while green circles are TFs. TFs|=|transcription factors.

**Figure 7.**
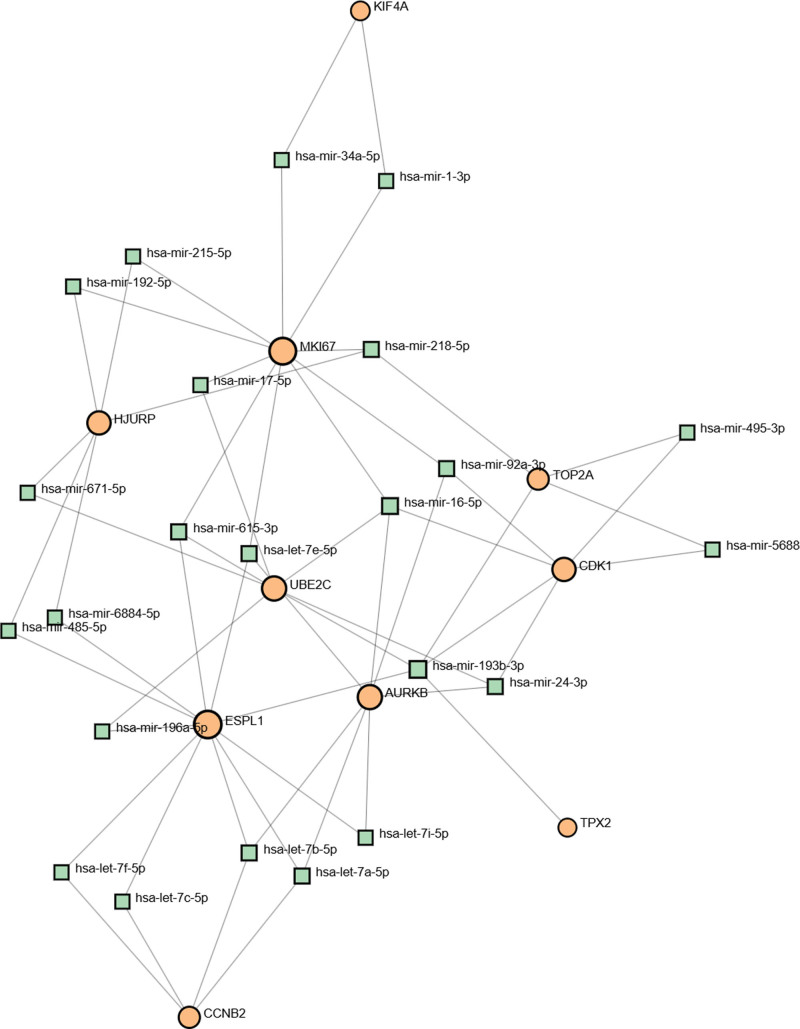
The construction of a regulatory interaction network for hub genes and miRNAs. In this figure, circle nodes represent hub genes and square nodes indicate miRNAs. mi-RNAs|=|micro-RNAs.

To identify substantial changes occurring at the transcriptional level and gain insight into hub genes, we used JASPAR, MiRTarBase, and NetworkAnalyst platforms to construct regulatory networks in which TFs and miRNAs bind to hub genes. The analysis results in Figures 6 and 7 showed that 42 TFs, including FOXC1, GATA2, YY1, and FOXL1, and 23 miRNAs such as hsa-miR-16-5p and has-miR-193b-3p, interacted with the hub genes.

### 3.5. Exploration of potential therapeutic drugs

The primary objective of this study is to identify drug candidates for the treatment of PC patients infected with SARS-CoV-2. By leveraging Enrichr and Drug Signatures Database, we narrowed down the top 10 molecules based on the *P* values of hub genes. These drugs include lucanthone, etoposide, troglitazone, resveratrol, calcitriol, ciclopirox, dasatinib, enterolactone, methotrexate, and irinotecan. Detailed information regarding these drug compounds is provided in Table 4.

**Table 4:**
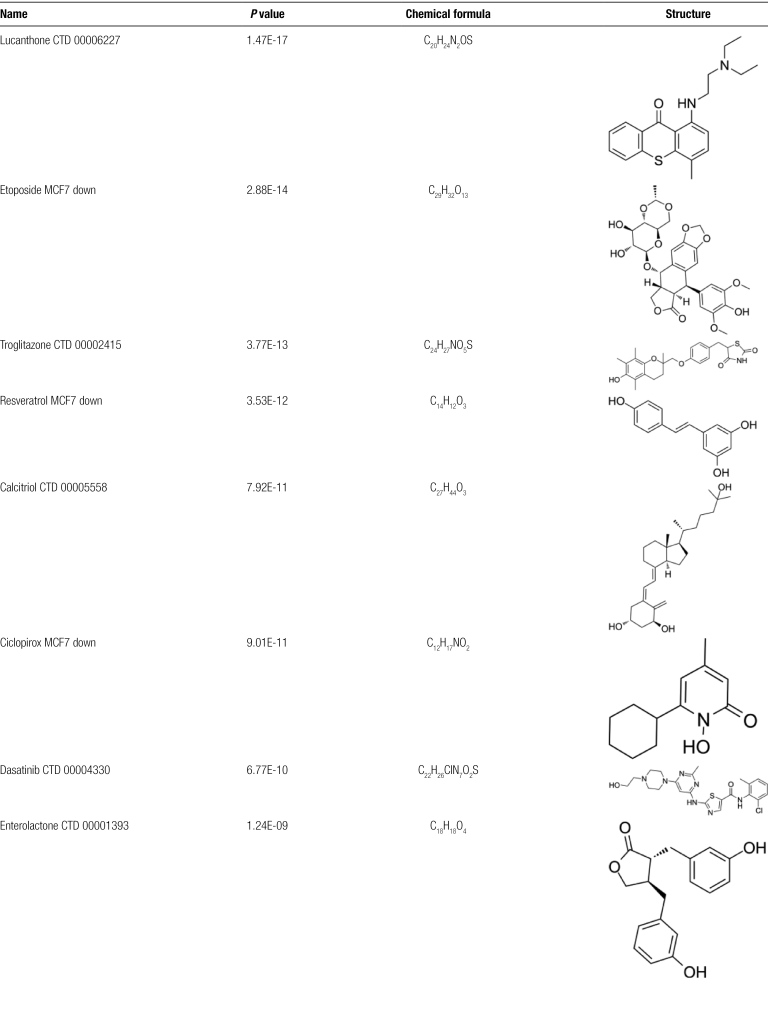
Potential drugs for COVID-19 and PC.

## 4. Discussion

The SARS-CoV-2 virus responsible for COVID-19 can worsen cancer progression. The virus’s interaction with ACE2 receptors, which are also found in pancreatic tissue, may impact tumor biology.^[12]^ COVID-19 can lead to severe immune dysregulation and cytokine storms, which can influence cancer progression. This altered immune environment may support tumor growth and metastasis.^[36]^ Patients with pancreatic cancer often have a weakened immune system due to the disease itself and treatments such as chemotherapy. This makes them more susceptible to COVID-19, which can lead to more severe symptoms and complications if they become infected.^[37]^ We performed a bioinformatics and systems biology analysis to uncover the relationship between COVID-19 and pancreatic cancer. The transcriptome analysis of patients with COVID-19 and PC identified 236 shared genes. Further in-depth analysis of these shared genes was conducted to explore the connection between COVID-19 and PC.

The PPI network of common DEGs was constructed using a STRING database to reveal potential associations between shared DEGs. The hub proteins in this network (ESPL1, HJURP, MKI67, KIF4A, CDK1, TOP2A, CCNB2, UBE2C, AURKB, and TPX2) are considered to be the most critical co-regulators of PC and SARS-CoV-2 infection. ESPL1 is a protein-coding gene that plays a key role in cell division.^[38]^ Overexpression or aberrant activation of ESPL1 can lead to aneuploidy and chromosomal instability, which are hallmarks of cancer. Research has shown that ESPL1 is involved in the development and progression of several types of cancer, including glioma,^[39]^ breast,^[40]^ stomach,^[41]^ bladder,^[42]^ liver,^[43]^ and endometrial^[44]^ cancers. HJURP is a protein-coding gene that plays a crucial role in centromere function and chromosomal stability by facilitating the assembly of the CENP-A nucleosome at centromeres.^[45]^ HJURP expression in pancreatic ductal adenocarcinoma (PDAC) cells and tissues is significantly higher compared to adjacent normal tissues, and high levels of HJURP are associated with poor survival rates. HJURP enhances the viability, spheroid formation, migration, and invasion of PDAC cells in vitro, and it also promotes tumor growth and metastasis in vivo. Mechanistically, HJURP mediates malignant behavior in PDAC through the MDM2/p53 axis, regulating MDM2 expression via H3K4me2 modification. Therefore, HJURP represents a promising biomarker and potential therapeutic target for PDAC prognosis and treatment.^[46]^ A systems biology analysis revealed that HJURP could be used to develop therapeutic interventions for COVID-19.^[47]^ MKI67 is a protein-coding gene that is highly expressed in proliferating cells and serves as a well-established marker for cell proliferation, playing a crucial role in both normal cellular growth and cancer development.^[48]^ MKI67 is associated with the activation of the NLRP3 inflammasome and can be used as a potential marker to identify patients with severe COVID-19.^[49]^ KIF4A is a protein-coding gene that encodes a kinesin family motor protein essential for chromosome condensation, segregation, and cytokinesis during cell division, thereby maintaining genomic stability.^[50]^ Reduced H3K27me3 modification at the KIF4A promoter sequence and decreased expression of the zeste cognate-2 enhancer (EZH2) increase KIF4A expression, contributing to the development of pancreatic cancer.^[51]^ A bioinformatics analysis revealed KIF4A as a key biomarker for COVID-19.^[52]^ CDK1 is a protein-coding gene that regulates the cell cycle by controlling the transition from the G2 phase to the M phase, playing a pivotal role in cell division and proliferation.^[53]^ Pharmacological or genetic inactivation of CDK1 not only blocks Jun-dependent immune checkpoint expression but also triggers histone-dependent immunogenic cell death in cancer cells in response to IFN-γ. This dual mechanism converts an immunologically “cold” tumor microenvironment into a “hot” one, significantly improving overall survival in various mouse models of pancreatic tumors (subcutaneous, in situ, and transgenic models). Additionally, abnormal expression of CDK1 and IDO1 is linked to poor survival in patients with several cancer types, including PDAC.^[54]^ CDK1 mediates human telomerase reverse transcriptase, and phosphorylation reveals RNA-dependent RNA polymerase activity, which is essential for transcription and replication of COVID-19 virus RNA.^[55]^ CDK1 may be up-regulated in critically ill patients, and researchers have also found that RNA-dependent RNA polymerase inhibitors have a positive therapeutic effect on COVID-19 patients.^[56]^ TOP2A is a protein-coding gene that encodes an enzyme critical for DNA replication, transcription, and chromosome segregation, playing a vital role in cell proliferation and genomic stability.^[57]^ Compared to nontumor tissues, the upregulation of TOP2A in pancreatic cancer is significantly associated with tumor metastasis and shorter survival in patients. TOP2A functions as a co-activator of β-catenin, promoting the epithelial-mesenchymal transition process and driving the malignant progression of pancreatic cancer.^[58]^ The role of TOP2A in COVID-19 has been partially reported, showing upregulation in blood cells of COVID-19 patients and indicating its potential as a therapeutic target.^[59]^ CCNB2 is a protein-coding gene that plays a crucial role in cell cycle regulation by controlling the transition from the G2 phase to mitosis, essential for cell division and proliferation.^[60]^ It has been reported that CCNB2 is not conducive to overall survival and relapse-free survival of pancreatic cancer and may be a key gene in the development of pancreatic cancer.^[61]^ CCNB2 is a crucial hub-shared gene in both silicosis and COVID-19, as well as a key hub-shared gene in COVID-19 and lung adenocarcinoma.^[62]^ UBE2C is a protein-coding gene that plays a vital role in the regulation of the cell cycle by targeting proteins for degradation via the ubiquitin-proteasome pathway, and its overexpression is associated with various cancers and poor prognosis.^[63]^ UBE2C enhances the expression of matrix metalloproteinases by activating the PI3K-Akt pathway. Additionally, UBE2C has been shown to bind to the epidermal growth factor receptor, stabilizing it and further activating the PI3K-Akt pathway, thereby promoting the progression of pancreatic cancer.^[64]^ A transcriptome sequencing study of peripheral blood of patients with pneumonia found that UBE2C expression was higher in patients with severe pneumonia than in patients with mild pneumonia.^[65]^ AURKB is a protein-coding gene that plays a crucial role in chromosome alignment, segregation, and cytokinesis during mitosis. Activation of AURKB induces the development of pancreatic cancer.^[66]^ AURKB has been identified as a DEG of SARS-CoV-2 in Caco-2 cells.^[67]^ TPX2 is a protein-coding gene crucial for spindle assembly and accurate chromosome segregation during mitosis, thereby playing an essential role in cell division and maintaining genomic stability.^[68]^ The expression of TPX2 is significantly higher in pancreatic tumors compared to normal tissues. Small interfering RNA targeting TPX2 can effectively reduce the growth of pancreatic cancer cells in vitro and induce apoptosis.^[69]^ TPX2 also shows potential as a novel intervention target and biomarker for COVID-19.^[47]^ As an antigenic component of a polyvalent recombinant fusion protein prophylactic vaccine, TPX2 promotes the production of high-titer antigen-specific antibodies and their isotypes. Animals vaccinated with the TPX2 antigen secrete higher levels of blood IFN-γ and demonstrate better immune protection compared to unvaccinated animals.^[70]^

Transcription factors and miRNAs that potentially act as upstream regulators of these hub genes were identified, providing deeper insights into the pathological mechanisms underlying these diseases. Determining potential drug compounds is the most critical part of this research. Several chemicals and drugs have been explored as potential therapeutics against both COVID-19 and pancreatic cancer. Lucanthone is an antischistosomal and anticancer agent that has shown potential in sensitizing tumors to radiation therapy and inhibiting the DNA repair process in cancer cells.^[71]^ Luanthrone enhances tumor necrosis factor-related apoptosis-inducing ligand apoptosis by down-regulating miR-216a-5p and DUB3-dependent Mcl-1 in human renal carcinoma cells to up-regulate DR5.^[72]^ Luanthrone has been reported as a potential treatment for COVID-19.^[73]^ Etoposide is a semisynthetic podophyllotoxin derivative widely used as chemotherapy for pancreatic cancer.^[74]^ Etoposide, a TOP II inhibitor, hinders the intracellular replication of SARS-CoV-2 structural proteins and mitigates the cytokine storm associated with COVID-19.^[75]^ Troglitazone can inhibit the growth of human pancreatic cancer cells by inhibiting MEK1/2-ERK1/2 signaling pathway.^[76]^ It has been discovered that troglitazone inhibits the SARS-CoV-2 NSP9 protein, which is essential for the virus’s replication.^[77]^ Resveratrol inhibited the activation of NFκB, thereby slowing the tumorigenesis of pancreatic cancer.^[78]^ Due to its antithrombotic and anti-inflammatory properties, resveratrol is anticipated to reduce mortality associated with COVID-19.^[79]^ Calcitriol is an active vitamin D3, also known as 1, 25-dihydroxyvitamin D3. Vitamin D receptor-mediated stromal reprogramming suppresses pancreatitis and enhances pancreatic cancer therapy.^[80]^ Calcitriol improves oxygenation in hospitalized patients with COVID-19.^[81]^ Ciclopirox is a broad-spectrum antifungal drug commonly used to treat fungal skin infections. The antifungal drug ciclopirox ethanolamine has been reported to be superior to gemcitabine in a pancreatic cancer model.^[82]^ In line with this research, Zhang et al^[83]^ identified ciclopirox as a potential therapeutic agent for treating patients with SARS-CoV-2 infection using drug prediction and simulated docking patterns. Dasatinib can enhance the inhibitory activity of paclitaxel and gemcitabine on human pancreatic cancer cells.^[84]^ Dasatinib treatment reduces SARS-CoV-2-related mortality in mice.^[85]^ Enterolactone, a lignan metabolite derived from plant-based foods, exhibits antioxidant and anti-inflammatory properties and is associated with reduced risks of hormone-related cancers. It exerts antitumor effects by regulating gene expression related to cell proliferation and the cell cycle, and by inhibiting the FAK/paxillin signaling pathway.^[86]^ Additionally, transcriptome analysis suggests that enterolactone could be a potential candidate for COVID-19 treatment.^[87]^ Methotrexate is a folate antagonist widely used to treat various cancers and inflammatory diseases, including pancreatic cancer.^[88]^ During the COVID-19 pandemic, methotrexate remains a viable treatment option for patients needing immunosuppressive therapy.^[89]^ Irinotecan, a chemotherapy drug used primarily for pancreatic cancer, has been investigated for its potential to mitigate COVID-19 symptoms by targeting viral replication mechanisms and modulating immune responses.^[36]^

It is important to acknowledge that this study has certain limitations. First, due to biases in information and methods, computational biology techniques cannot fully recapitulate the underlying genetic linkages. Second, we currently lack in vivo or in vitro experiments to validate these findings, with specific experiments planned for future steps. Lastly, there remains a significant challenge in translating experimental results into clinical applications.

## 5. Conclusion

To explore the common relationship between SARS-CoV-2 infection and pancreatic cancer, we utilized transcriptomic data analysis to identify shared pathways and biomarkers. Bioinformatics tools revealed 236 common differentially expressed genes in PC and COVID-19. GO terms and signaling pathway enrichment analysis showed that these DEGs are mainly involved in viral genome replication and tumorigenesis pathways. Additionally, protein–protein interaction (PPI) analysis identified the top 10 hub genes: ESPL1, HJURP, MKI67, KIF4A, CDK1, TOP2A, CCNB2, UBE2C, AURKB, and TPX2, which may serve as therapeutic targets for both COVID-19 and PC. Further analysis of gene-TFs and gene-miRNAs associations provided deeper insights into COVID-19 progression. Potential drugs identified for treating COVID-19 patients with PC include lucanthone, etoposide, troglitazone, resveratrol, calcitriol, ciclopirox, dasatinib, enterolactone, methotrexate, and irinotecan. This research offers a foundation for further investigation into the molecular mechanisms and treatment strategies for COVID-19 and PC.

## Acknowledgments

The authors acknowledge all patients who donate samples, the contributors who upload datasets, and the GEO database for providing platforms.

## Author contributions

**Investigation:** Chengxiang Fang, Haiyan Sun.

**Methodology:** Chengxiang Fang.

**Project administration:** Chengxiang Fang.

**Resources:** Chengxiang Fang.

**Visualization:** Haiyan Sun, Dongsheng Zhai.

**Data curation:** Jing Wen, Xuehu Wu.

**Supervision:** Jing Wen.

**Validation:** Jing Wen, Qian Wu, Dongsheng Zhai.

**Writing—original draft:** Jing Wen, Qian Wu, Dongsheng Zhai.

**Conceptualization:** Xuehu Wu, Qian Wu.

**Formal analysis:** Xuehu Wu.

**Writing—review & editing:** Xuehu Wu, Qian Wu, Dongsheng Zhai.

**Funding acquisition:** Qian Wu, Dongsheng Zhai.
